# Trends and drivers of anthropogenic NO_*x*_ emissions in China since 2020

**DOI:** 10.1016/j.ese.2024.100425

**Published:** 2024-04-27

**Authors:** Hui Li, Bo Zheng, Yu Lei, Didier Hauglustaine, Cuihong Chen, Xin Lin, Yi Zhang, Qiang Zhang, Kebin He

**Affiliations:** a Shenzhen Key Laboratory of Ecological Remediation and Carbon Sequestration, Institute of Environment and Ecology, Tsinghua Shenzhen International Graduate School, Tsinghua University, Shenzhen 518055, China; b State Environmental Protection Key Laboratory of Sources and Control of Air Pollution Complex, Beijing 100084, China; c State Environmental Protection Key Laboratory of Environmental Planning and Policy Simulation and Center of Air Quality Simulation and System Analysis, Chinese Academy of Environmental Planning, Beijing 100041, China; d Laboratoire des Sciences du Climat et de l’Environnement, LSCE/IPSL, CEA-CNRS-UVSQ, Université Paris-Saclay, Gif-sur-Yvette, France; e Center for Satellite Application on Ecology and Environment, Ministry of Ecology and Environment of China, Beijing 100094, China; f Institute of Future Human Habitats, Tsinghua Shenzhen International Graduate School, Tsinghua University, Shenzhen 518055, China; g Ministry of Education Key Laboratory for Earth System Modeling, Department of Earth System Science, Tsinghua University, Beijing 100084, China; h State Key Joint Laboratory of Environment Simulation and Pollution Control, School of Environment, Tsinghua University, Beijing 100084, China

**Keywords:** China's NO_*x*_ emissions, Pollution control, Socio-economic drivers, Atmospheric inversion

## Abstract

Nitrogen oxides (NO_*x*_), significant contributors to air pollution and climate change, form aerosols and ozone in the atmosphere. Accurate, timely, and transparent information on NO_*x*_ emissions is essential for decision-making to mitigate both haze and ozone pollution. However, a comprehensive understanding of the trends and drivers behind anthropogenic NO_*x*_ emissions from China—the world's largest emitter—has been lacking since 2020 due to delays in emissions reporting. Here we show a consistent decline in China's NO_*x*_ emissions from 2020 to 2022, despite increased fossil fuel consumption, utilizing satellite observations as constraints for NO_*x*_ emission estimates through atmospheric inversion. This reduction is corroborated by data from two independent spaceborne instruments: the TROPOspheric Monitoring Instrument (TROPOMI) and the Ozone Monitoring Instrument (OMI). Notably, a reduction in transport emissions, largely due to the COVID-19 lockdowns, slightly decreased China's NO_*x*_ emissions in 2020. In subsequent years, 2021 and 2022, reductions in NO_*x*_ emissions were driven by the industry and transport sectors, influenced by stringent air pollution controls. The satellite-based inversion system developed in this study represents a significant advancement in the real-time monitoring of regional air pollution emissions from space.

## Highlights

• We integrate top-down inversion with bottom-up information to infer NO_*x*_ emissions.
• China's monthly and sectoral NO_*x*_ emissions are estimated from 2020 to 2022.
• China's NO_*x*_ emissions have declined since 2020 despite increased fuel consumption.
• Air pollution control led to widespread emission declines in 2021 and 2022.

## Graphical abstract

**Figure:**
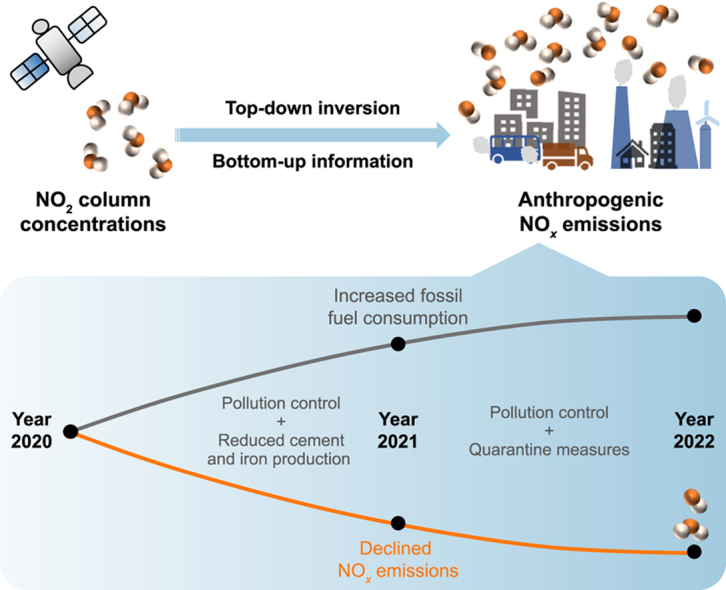

## 1 Introduction

Nitrogen oxides (NO_*x*_ = NO + NO_2_), an active and short-lived air pollutant, have been well-known for their crucial role in ozone photochemistry [1], haze chemistry [2], and climate impacts [3]. The emissions of NO_*x*_ contribute to the formation of acid rain [4], O_3_ pollution [5], and haze pollution [6], leading to air pollution-related deaths [7] and damage to ecosystems. China is the largest emitter of NO_*x*_ at present, accounting for about 24% of the global total at present (https://edgar.jrc.ec.europa.eu/) [8]. Recognizing the NO_*x*_-induced environmental issues, China has been reinforcing the control of NO_*x*_ emissions from anthropogenic sources, especially since 2013, when severe haze attracted broad attention and triggered the toughest-ever air pollution control actions [9].

The major sources of NO_*x*_ emissions are fossil fuel consumption in power generation [[10], [11], [12]], transportation [13], iron and steel production [14], and cement production [15]. China has enforced ultra-low emission standards for coal-fired power plants [16,17] as well as cement and iron production [18]. Emission standards for on-road vehicles have been progressively upgraded from China III to China VI from 2017 to 2023, imposing stricter limits on NO_*x*_ emissions standards from 150 to 60 mg km^−1^ [19]. Measures have also been taken to phase out small or outdated industrial capacities, coal-fired power plants, and old “yellow-labeled” vehicles. These efforts together have led to a steady decline in China's NO_*x*_ emissions since 2012, with the industry, transport, and power sectors as primary contributors [13,20,21].

Since 2020, the world has experienced drastic socio-economic changes, including global economic inflation [22], pandemic prevention measures [23], and initiatives toward carbon neutrality [24], which have incurred widespread and substantial changes in human activities. These socio-economic changes have introduced substantial challenges in evaluating anthropogenic emission changes timely and the associated impacts on the atmospheric environment. The conventional bottom-up approach, based on activity data and emission factors, is limited by the availability of timely and accurate data [25]. The top-down method, using atmospheric observation constraints to infer emissions, can circumvent the dilemma of data availability, as most observational data and meteorological reanalysis fields are publicly accessible and updated in near real-time [26,27]. Satellite-based monitoring has become a widely used tool for NO_*x*_ emission estimation, benefiting from its large signal-to-noise ratio, broad spatial coverage and high spatial resolution, and continuous temporal availability [[28], [29], [30], [31]].

A decline in anthropogenic NO_*x*_ emissions was temporally observed in 2020, driven by the strict quarantine measures [32]. However, we currently lack a complete understanding of the changes and drivers of China's anthropogenic NO_*x*_ emissions after 2020. To bridge the data gap, we utilize our previously developed and well-validated atmospheric inversion system based on satellite observations [33,34] to estimate the monthly and sectoral NO_*x*_ emissions in the Chinese mainland from 2020 to 2022. To confirm the robustness of our results**,** we utilize TROPOspheric Monitoring Instrument (TROPOMI) and Ozone Monitoring Instrument (OMI) satellite NO_2_ retrievals as observational constraints in our inversions, respectively, as mutual verification. China's anthropogenic NO_*x*_ emissions by month, sector, and province are then further investigated in detail to resolve the drivers of emission variations, including air pollution control and socio-economic factor changes.

## 2 Materials and methods

### 2.1 Satellite NO_2_ retrievals

TROPOMI, on board the European Copernicus Sentinel-5 Precursor (S5P) launched in October 2017 [35], is the most widely used satellite instrument monitoring NO_2_ pollution because it offers global daily NO_2_ tropospheric vertical column densities (TVCDs) sampled at 13:30 local time with a current resolution of up to 5.5 × 3.5 km [36]. Here we used version 2.4.0 of TROPOMI NO_2_ TVCDs as the observational constraint in inversion (https://www.temis.nl/airpollution/no2col/no2regio_tropomi.php), which could reduce the low-biased errors of the previous data versions [37]. To evaluate our results, we also employed OMI NO_2_ retrievals version 3 [38] as observational constraints in our inversion separately. OMI is on the National Aeronautics and Space Administration (NASA)'s EOS-Aura spacecraft, providing global daily NO_2_ TVCDs sampled at 13:40 local time with a resolution of 13 × 24 km (https://aura.gesdisc.eosdis.nasa.gov/data/Aura_OMI_Level2/OMNO2.003/) [39]. We filtered out the data of poor quality for TROPOMI and OMI [40] and mapped the NO_2_ TVCDs at a resolution of 0.5° × 0.625° to match the horizontal resolution of our chemical transport model [33]. Our analysis only retained the grid cells dominated by anthropogenic NO_*x*_ emissions, where the daily NO_2_ TVCDs exceeded 1 × 10^15^ molecules cm^−2^ [41].

### 2.2 Prior NO_*x*_ emissions

We used the bottom-up approach to estimate China's NO_*x*_ emissions between 2020 and 2022 as prior, based on the MEIC (Multi-resolution Emission Inventory for China) emission inventory in 2019 and the year-on-year monthly changes in activity levels from 2019 to the target years (i.e., 2020, 2021, and 2022) [32]. The monthly changes in thermal power generation, cement production, iron production, and manufacturing value added from the National Bureau of Statistics (http://www.stats.gov.cn/) were used to infer the changes in activity levels of the power, cement, iron, and other industries, respectively. To account for January and February separately, combined in official statistical reports, we utilized the production index to differentiate the activity levels between these two months. Regarding the transport sector, we employed monthly changes in on-road freight turnover and construction area to represent the changes in emissions from the on- and off-road sectors. The changes in the freight turnover served as a good proxy for heavy-duty truck activities, which accounted for a majority of transport NO_*x*_ emissions in China [42]. For the residential heating emissions, the provincial population-weighted heating degree days were used to represent the monthly changes in activity levels of heating boilers and stoves, except for provinces located on the same latitude band as Guangdong and those to the south (i.e., Guangdong, Guangxi, and Hainan provinces), where residential stoves were rarely used. The residential heating NO_*x*_ emissions were low and assumed unchanged since 2019 [34].

### 2.3 Inversion estimation of sectoral NO_*x*_ emissions

The monthly total NO_*x*_ emissions between 2020 and 2022 in China were inferred from satellite retrievals of NO_2_ TVCDs using the mass balance method [43], assuming a localized relation between the changes in NO_2_ TVCDs and NO_*x*_ emissions.

(1) $$E_{t,i,\text{sate},y}={(1+\beta_{t,i}\left(\frac{\Delta \Omega }{\Omega }\right)}_{t,i,\text{anth},y})\times E_{t,i,\text{bottom}-\text{up},2019}$$

(2) $${\left(\frac{\Delta \Omega }{\Omega }\right)}_{t,i,\text{anth},y}=\frac{\Omega_{t,i,\text{sate},y}}{\Omega_{t,i,\text{sate},2019}}-\frac{\Omega_{t,i,\text{simu}\_\text{fixemis},y}}{\Omega_{t,i,\text{simu},2019}}$$

In these equations, *t*, *i*, and *y* represent the month, grid cell (i.e., 0.5° × 0.625°), and year (i.e., 2020, 2021, and 2022), respectively. *E*_*t,i,*sate*,y*_ is the anthropogenic NO_*x*_ emissions constrained by satellite NO_2_ TVCDs (i.e., TROPOMI or OMI). *E*_*t,i,*bottom-up,2019_ is the anthropogenic NO_*x*_ emissions in 2019 from the MEIC model. *β*_*t,i*_ is a unitless factor relating the changes in NO_2_ TVCDs to the changes in anthropogenic NO_*x*_ emissions [44], acquired by a perturbation (i.e., −40%) of China's anthropogenic NO_*x*_ emissions in 2019 (details in Materials and Methods in the Supplementary Information (SI)). For abnormal occurrences of *β*_*t,i*_, specifically when *β*_*t,i*_ exceeds 2, we masked those grids to mitigate the influence of these anomalies on the results. ${\left(\frac{\Delta \Omega }{\Omega }\right)}_{t,i,\text{anth},y}$ refers to the relative changes in satellite NO_2_ TVCDs (*Ω*) due to anthropogenic NO_*x*_ emissions from 2019 to year *y*. $\frac{\Omega_{t,i,\text{sate},y}}{\Omega_{t,i,\text{sate},2019}}$ indicates the relative differences in satellite NO_2_ TVCDs, and $\frac{\Omega_{t,i,\text{simu}\_\text{fixemis},y}}{\Omega_{t,i,\text{simu},2019}}$ represents the relative differences in NO_2_ TVCDs caused by meteorological fluctuations, estimated by GEOS-Chem model simulations with the fixed 2019 emissions and meteorological field in year *y*. We used GEOS-Chem 12.3.0 (https://geoschem.github.io/) driven by the meteorological fields from the MERRA-2 Reanalysis of the NASA Global Modeling and Assimilation Office [45]. Details on the inversion method and model settings can be found in our previous work (Materials and Methods in SI) [33,34]. While the GEOS-Chem simulated NO_2_ TVCDs exhibit a low bias compared to the TROPOMI observation, their spatial distribution and annual means are in strong agreement (Fig. S1). Furthermore, adopting relative changes in either the simulated NO_2_ TVCDs or TROPOMI observational NO_2_ TVCDs (equation (2)) can alleviate the influence of the low bias in absolute NO_2_ TVCDs on emission estimates.

The monthly NO_*x*_ emissions were attributed to each source sector by integrating the prior information on sectoral emission spatial distributions. We summarized the difference between inversion and prior emission estimates in grid cells dominated by each source sector, whose contribution exceeded 50% of the grid's total emissions in prior. These discrepancies were used to derive scaling factors for each source sector and correct the prior NO_*x*_ emissions by sector. The corrected prior emissions were finally rescaled to ensure consistency with the satellite-constrained NO_*x*_ emissions for national totals. Details for sectoral emission estimation can be found in our previous studies (Materials and Methods in SI) [33,34]. The good correlation observed between GEOS-Chem simulated and TROPOMI observational NO_2_ TVCDs (Fig. S1), along with the correlation between GEOS-Chem simulated and ground station monitored surface NO_2_ concentrations (http://www.cnemc.cn/) (Fig. S2), attests to the robustness and reliability of our NO_*x*_ emission estimations.

## 3 Results and discussion

### 3.1 Decline in China's NO_*x*_ emissions since 2020

**Fig. 1:**
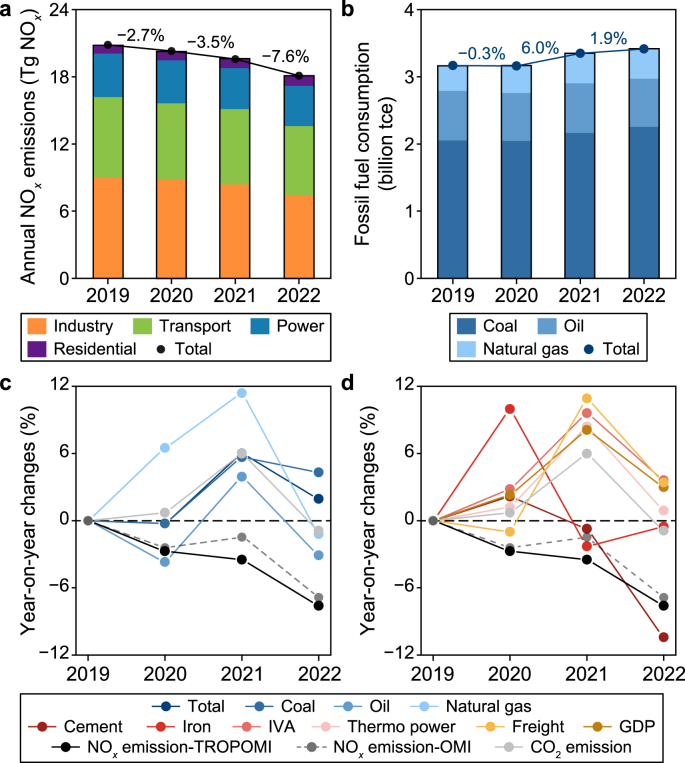
Changes in TROPOMI-constrained annual NO_*x*_ emissions, fossil fuel consumption, and main anthropogenic activities between 2020 and 2022. **a**, The changes in annual NO_*x*_ emissions between 2020 and 2022. **b**, The annual changes in fossil fuel consumption between 2020 and 2022, including coal, oil, and natural gas. **c**, Year-on-year changes in fossil fuel consumption, NO_*x*_, and CO_2_ emissions between 2020 and 2022. **d**, Year-on-year changes in social-economic activities (http://www.stats.gov.cn/), NO_*x*_, and CO_2_ emissions between 2020 and 2022. The changes in CO_2_ emissions here are the averages of the estimates from Carbon Monitor (https://cn.carbonmonitor.org/) and IEA (https://www.iea.org/). Fossil fuel consumption refers solely to the quantity consumed through combustion. IVA: industry value added.

Our inversions reveal that China's NO_*x*_ emissions have declined since 2020, with a progressively steeper rate of decrease (black curves in Fig. 1). The year-on-year reductions in China's NO_*x*_ emissions, as monitored by the TROPOMI satellite, were 2.7% in 2020, 3.5% in 2021, and 7.6% in 2022 (Fig. 1a), which align with the OMI-constrained inversion (dashed curves in Fig. 1c and d). China's NO_*x*_ emissions are estimated to have declined by 10.8% from 2020 to 2022, which is consistent with the surface NO_2_ concentrations measured by ground stations in China (down by 12.5% from 2020 to 2022) (details refer to Section 1.7 in Materials and Methods in SI). Besides, we utilize the TROPOMI-constrained NO_*x*_ emissions to drive the global chemical transport model (LMDZ-INCA) used by Peng et al. [46]. The simulated NO_2_ TVCDs based on LMDZ-INCA closely replicate the annual variations observed by TROPOMI (deep orange line in Fig. S3b). The broad consistency of multiple lines of evidence confirms the rapid drop in China's NO_*x*_ emissions since 2020. Unless stated separately, the inversion NO_*x*_ emissions in the following text refer to the TROPOMI-based estimates.

Industrial production, energy use, and CO_2_ emissions in China did not decline as the inversion-based NO_*x*_ emissions but increased moderately since 2020 (Fig. 1b–d). The thermal power generation and industry value added grew annually by 0.9–8.4% and 2.8–9.6%, respectively, between 2020 and 2022. The freight turnover decreased by 1.0% in 2020 but rebounded by 10.9% in 2021 and 3.4% in 2022, corresponding to rapid economic growth. Heavy industries in China showed fluctuations in their annual production, with iron and cement production rising in 2020 but dipping in 2021 and 2022. The total consumption of fossil fuels in China, including coal, oil, and natural gas, slightly decreased in 2020 by 0.3% but kept increasing since 2021, with growth rates of 6.0% in 2021 and 1.9% in 2022. China's CO_2_ emissions, averaged between Carbon Monitor [47] and International Energy Agency (IEA 2023, https://www.iea.org/) estimates, increased by 0.7% and 6.0% in 2020 and 2021, respectively, and decreased slightly by 0.9% in 2022 (grey solid curves in Fig. 1c and d).

The discrepancy between NO_*x*_ and CO_2_ emission variations since 2020 reflects the divergent changes in the source sectors with different NO_*x*_-to-CO_2_ emission ratios or the influences from NO_*x*_ pollution control. To disentangle the possible drivers, we estimate China's NO_*x*_ emissions by combining the sectoral NO_*x*_ emissions in 2019 from MEIC with the annual percentage changes in sectoral CO_2_ emissions since 2019 from Carbon Monitor (Fig. S3a). We further utilize such simply updated NO_*x*_ emissions (refer to Carbon Monitor-based NO_*x*_ emissions hereinafter) to drive the LMDZ-INCA model [46]. This simulated NO_2_ TVCDs presented a 3.6% decline over China in 2020 compared to 2019, consistent with the TROPOMI observations (Fig. S3b). The Carbon Monitor-based NO_*x*_ emissions reduction in 2020 agrees with our inversion results, in contrast to the slight growth of Carbon Monitor's CO_2_ emissions. This suggests that the sectors with large NO_*x*_-to-CO_2_ emission ratios decreased their emissions while the others increased emissions, causing an opposite change in total NO_*x*_ and CO_2_ emissions in 2020. The transport sector has the largest NO_*x*_-to-CO_2_ emission ratio in China (0.008, 0.002, 0.001, and 0.001 for transport, industry, power, and residential sectors according to the 2019 MEIC emission inventory) [13]. Their emissions possibly dropped in 2020, as reflected by the reduction in oil consumption (Fig. 1c, Fig. S4, Tables S1 and S2) and freight turnover (Fig. 1d).

The NO_*x*_ emissions from the Carbon Monitor-based estimates increase by 7.6% in 2021 and decrease by 3.0% in 2022, which are close to the Carbon Monitor CO_2_ emission changes, while our inversion-based NO_*x*_ emissions show substantial declines (Fig. S3a). The atmospheric simulation of LMDZ-INCA driven by the Carbon Monitor-based NO_*x*_ emissions shows a 5.3% increase in NO_2_ TVCDs over China in 2021, while TROPOMI and OMI observations reveal a decrease of 3.9% and 2.1%, respectively, suggesting that China's NO_*x*_ emissions did not rebound like CO_2_ emissions (Fig. S3b). Although the changes in activity levels of different source sectors tend to result in concurrent interannual variations of NO_*x*_ and CO_2_ emissions, the other factors, most likely the NO_*x*_ pollution control related to clean air actions, drive down NO_*x*_ emissions fast.

### 3.2 Drivers of NO_*x*_ emissions decline in China

**Fig. 2:**
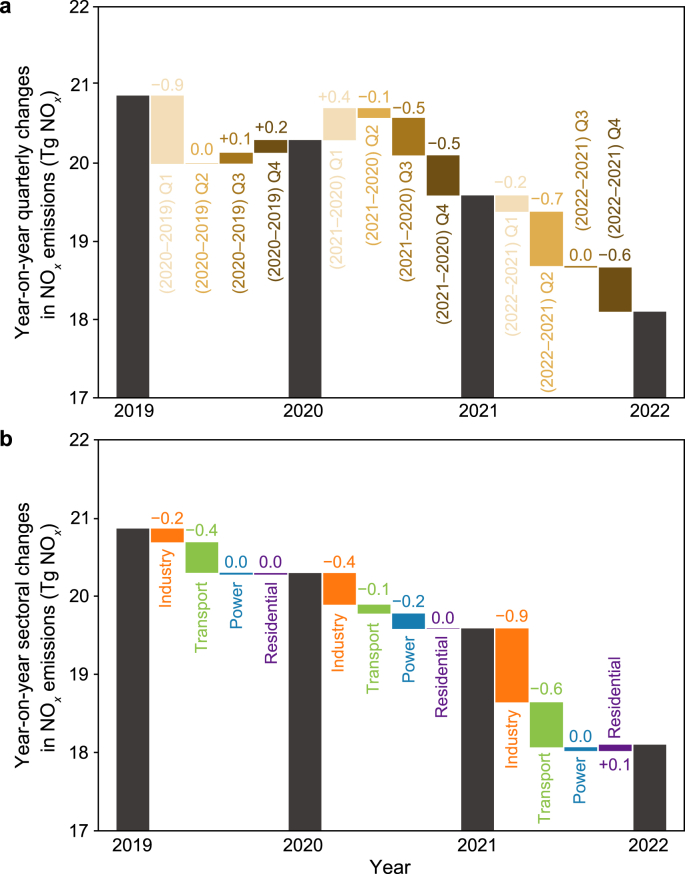
Year-on-year NO_*x*_ emission variations by quarter (**a**) and sector (**b**) between 2020 and 2022.

We break out the annual reductions in inversion-based NO_*x*_ emissions since 2020 by quarter (Fig. 2a) and by source sector (Fig. 2b) to illustrate the socio-economic drivers. Our inversion constrained by OMI observations (Fig. S5) provides consistent results as TROPOMI. In 2020, the analysis of emission variation by quarter and sector suggests that China's NO_*x*_ emissions declined in the first quarter (Q1) while rebounded in the remaining three-quarters (Fig. 2a). This is mainly due to the stringent lockdown response to the first wave of the Coronavirus Disease 2019 (COVID-19) pandemic in China lasting from January 23 to April 7 in 2020 and the rapid emissions rebound after that when the lockdown restriction lifted [33,48]. The transport sector, which was largely influenced by the COVID-19 lockdown, accounted for two-thirds of the annual emissions decline in 2020 (Fig. 2b). Besides, the transition from traditional vehicles to electric cars, promoted by the Chinese government, has further contributed to the reduction of NO_*x*_ emissions in the transport sector [49]. This is consistent with our analysis, which identifies the sector with large NO_*x*_-to-CO_2_ emission ratios as one of the main factors driving down China's NO_*x*_ emissions in 2020.

In 2021 and 2022, China's NO_*x*_ emissions declined in all quarters except Q1 2021, compared to the corresponding quarter of the year before (Fig. 2a). The emissions increase in Q1 2021 is because of the lockdown-induced emissions drop in Q1 2020. The industry sector primarily dominated the emissions decline in 2021, while the industry and transport sectors both drove the decline in 2022 (Fig. 2b). The NO_*x*_ pollution control in these two sectors is probably the main driver of the decline in emissions. China had completed the ultra-low emission retrofitting for 145 million tons and 250 million tons of steel production capacity, respectively, as of 2021 and 2022. Pollution control endeavors were also pursued in the cement, coke, and petrochemical industries (https://www.mee.gov.cn/ywdt/zbft/202303/t20230328_1022381.shtml). The transport sector has seen the enforcement of stricter vehicle emission standards, such as the China VI standard for light-duty vehicles that commenced in 2020 [50]. In 2021 and 2022, there was a total elimination of 4.2 million (https://www.mee.gov.cn/ywdt/hjywnews/202204/t20220421_975549.shtml) and 6.2 million (https://www.mee.gov.cn/ywdt/zbft/202303/t20230328_1022381.shtml) polluted and old vehicles, respectively.

Other factors, such as the structural change in China's economy and the COVID-19 influences, also contributed to the steady decline in NO_*x*_ emissions in 2021 and 2022. For example, the substantial decreases of NO_*x*_ emissions in the third quarter (Q3) and fourth quarter (Q4) of 2021 were concurrent with the drop in cement and iron production (Fig. S6), possibly due to the tightened regulation for the real estate sector since July 2021 (https://www.mohurd.gov.cn/). The NO_*x*_ emissions decline in 2022 mainly occurred in the second quarter (Q2) and Q4, when cement production declined by 16.7% and 5.1%, respectively, and the on-road freight turnover decreased by 1.3% and 2.5%, respectively. The emission decreases also coincided with the diminished economic activities due to the Omicron-incurred lockdown in Q2 [51] and the nationwide optimization of the COVID-19 policies at the end of 2022 (https://www.gov.cn/). Still, as shown in Section 3.1, air pollution control measures dominated China's NO_*x*_ emissions cut in 2021 and 2022.

### 3.3 NO_*x*_ emission changes and drivers by province

**Fig. 3:**
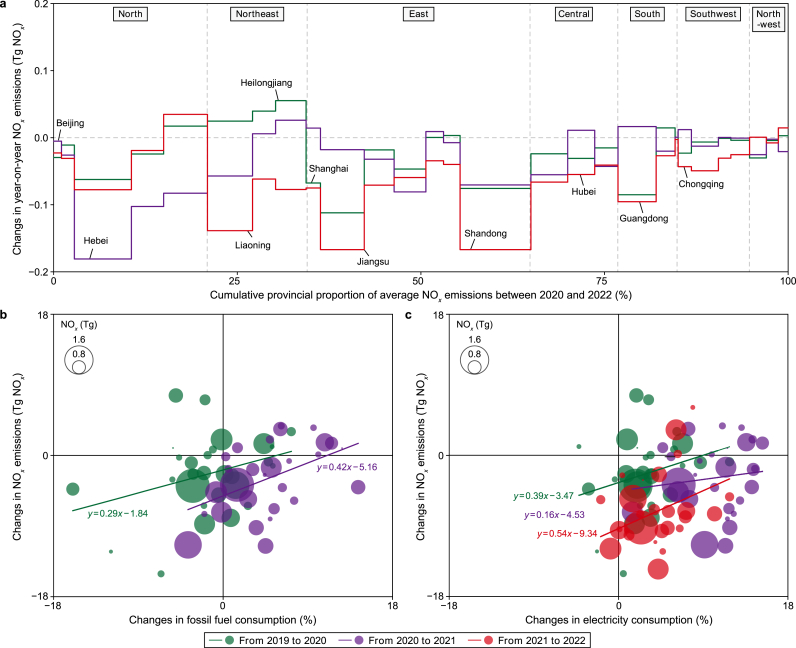
Provincial contribution to the year-on-year NO_*x*_ emission changes in China between 2020 and 2022. **a**, Provincial contribution to the year-on-year NO_*x*_ emission changes between 2019 and 2022 (region classification is introduced in Table S3). **b**, Correlations between the changes in provincial NO_*x*_ emissions and provincial fossil fuel consumption (refers only to the quantity consumed through combustion). Due to the unavailability of provincial fossil fuel consumption data for 2022, this panel only presents data from 2019 to 2020 and 2020 to 2021. **c**, Correlations between the changes in provincial NO_*x*_ emissions and provincial electricity consumption. The size of the dots refers to the provincial NO_*x*_ emissions.

The changes in China's NO_*x*_ emissions since 2020 vary by province and present broad spatial heterogeneity (Fig. 3a, Fig. S7), as well as uneven distribution of emissions and NO_2_ TVCDs (Figs. S7–S9). In 2020, when COVID-19 started to hit China, a majority of China's provinces observed a decline in both fossil fuel consumption and inversed NO_*x*_ emissions compared to those in 2019 (Fig. 3b). The impacts of the COVID-19 lockdowns on industry and transport sectors led to substantial emission reductions in the provinces with high industrialization levels and dense populations (Fig. S10). The provinces with large emission reductions were mostly located around Hubei, which experienced stringent lockdowns (Fig. S7a). For instance, Hubei, Shanghai, and Jiangsu witnessed NO_*x*_ emission reductions of 4.3%, 15.1%, and 8.7%, respectively. In contrast, Heilongjiang, Jilin, and Liaoning, the provinces in Northeast China that are geographically distant from Hubei, observed increases in NO_*x*_ emissions by 7.6%, 7.1%, and 2.0%, respectively, in 2020 (Fig. 3a, Fig. S7a). This rise in NO_*x*_ emission in these provinces aligns with their increase in industrial production. Taking industry value added as an example, there was a notable upswing of 3.3%, 6.9%, and 1.8% in Heilongjiang, Jilin, and Liaoning, respectively.

Since 2021, NO_*x*_ emissions have declined remarkably in most of China's provinces (Fig. 3a, Figs. S7b and c), despite the increased consumption of fossil fuel (Fig. 3b) and electricity (Fig. 3c), which reflects the nationwide efficacy of NO_*x*_ pollution control measures. Seasonal fluctuations in industrial production and the COVID-19 impacts further shaped different changes in NO_*x*_ emissions across provinces, modulated by the economic structure of each province. For example, in the latter half of 2021, iron production in Hebei — the province accounting for approximately one-quarter of China's total iron production — decreased by 17.5% compared to the corresponding period in 2020, which contributed to the 11.4% decline in Hebei's NO_*x*_ emissions in 2021 compared to those in 2020. Conversely, the provinces whose economic structure is dominated by the service industry, finance, and vehicle manufacturing, such as Shanghai, Guangdong, and Chongqing, increased NO_*x*_ emissions in 2021 due to the rebound in economic growth with few COVID-19 impacts.

In 2022, provinces with high levels of industrialization and populations reduced NO_*x*_ emissions substantially (Figs. S7c and S10), reflecting the combined impacts of air pollution control and socio-economic factor changes. Shanghai, Jiangsu, and Chongqing emerged as the top three provinces with the greatest NO_*x*_ emissions decline, down by 19.0%, 14.5%, and 12.3%, respectively. These developed, densely-populated regions were typically influenced more by the COVID-19 Omicron wave in 2022, and their emissions were slashed by lockdowns [52,53]. On the contrary, the provinces with fewer population densities in North and Northwest China increased emissions in 2022 (Fig. 3a, Fig. S7c), including Ningxia (+6.1%), Nei Mongol (+3.3%), and Shaanxi (+0.2%). Overall, the relationship between changes in provincial NO_*x*_ emissions and its economic structure was similar in 2020 and 2022, the two years with widespread COVID-19 lockdowns, suggesting consistent pandemic impacts on provincial NO_*x*_ emissions (Fig. S10).

### 3.4 Uncertainty and limitation

The primary source of uncertainty in our NO_*x*_ emission estimation arises from systematic and random errors associated with satellite NO_2_ retrievals [54], simulated NO_2_ columns from the GEOS-Chem model [55], and the inversion algorithm. While these errors may be inevitable, we have taken measures to minimize their potential influence on our conclusions as much as possible. To reduce systematic error influences stemming from satellite data, we employ relative changes in satellite NO_2_ as observational constraints in the inference of the total NO_*x*_ emission changes across different years, as indicated in equation (1). Likewise, we establish the relationship between the relative changes in NO_*x*_ emissions and NO_2_ columns (*β*) using the GEOS-Chem model, thereby reducing systematic errors stemming from the model simulations. Above all, our findings are based on spatial and sectoral aggregations to decrease random errors associated with the estimation process and the data used. Our previous studies have shown the insensitivity of estimated emissions to crucial parameters like *β* in the inversion algorithm, demonstrating the inversion results' robustness [33,34]. The NO_*x*_ emissions constrained by TROPOMI and OMI satellites exhibit comparable emission estimates since 2020, confirming the reliability of our results. Besides, the LMDZ-INCA simulated NO_2_ TVCDs using our inversion-based NO_*x*_ emissions closely match the interannual variations observed by satellites (deep orange line in Fig. S3), underscoring the reliability of the key findings in this study.

However, our methodology has certain limitations that warrant future improvements. First, we have simplified the nonlinear relationship between NO_*x*_ emissions and NO_2_ TVCDs and have not considered the inter-grid transport of NO_2_, using a linear relationship (*β*) in the current inversion system on a grid-by-grid (resolution of 0.5° × 0.625°) and month-by-month base. This assumption of approximate linearity and locality between NO_*x*_ emissions and NO_2_ TVCDs may introduce uncertainties, particularly during colder months when the NO_2_ lifetime is extended [56]. Second, the horizontal resolution affects the establishment of localized links between them, as grids with excessively fine resolution pose challenges related to inter-grid transport, while grids with overly coarse resolution hinder the attribution of sectoral emissions [57], necessitating a more agile and appropriate selection. Third, our focus is solely on anthropogenic NO_*x*_ emissions within grids where daily NO_2_ TVCDs exceed 1 × 10^15^ molecules cm^−2^, irrespective of the influence of specific events like wildfires [41]. While few wildfire emissions are compared to anthropogenic sources in China, exercising caution and proactively identifying specific regions affected by such events is essential. This precautionary measure is necessary to mitigate the potential impact of the increasingly frequent wildfires expected in the future due to climate change [58,59].

## 4 Conclusions

This study reveals a steady decline in China's NO_*x*_ emissions from 2020 to 2022, marking a reduction of 2.7% in 2020, 3.5% in 2021, and 7.6% in 2022, as confirmed by satellite-based atmospheric inversions. The reduced transport emissions, mainly due to the COVID-19 lockdown, led to the NO_*x*_ emissions decline in 2020 and contributed 68.9% to the overall reduction. The industry and transport sectors drove down China's NO_*x*_ emissions in 2021 and 2022 on a larger scale, which accounted for over 70% of the total reduction, possibly caused by air pollution control, as per our information. This study examines the encouraging outcome of China's ongoing clean air measures, which offers a viable blueprint for other countries grappling with their air quality challenges. It is noteworthy that, in the context of this study revealing the driving force of pollution control in reducing NO_*x*_ emissions, the rapid rebound in China's NO_2_ concentration in early 2023, as we previously disclosed, further confirms the rapid resurgence of economic and human activities over a relatively short period can still offset the effects of air pollution control temporarily [40]. Despite a decrease in NO_*x*_ emissions, we have not observed a concurrent decline in China's CO_2_ emissions due to the increased fossil fuel consumption, which suggests a difficulty in achieving coordinated governance of air quality and climate pollutants under the current energy structure. We need effective tools to explore and support the energy, climate, and air quality policy synergies. The satellite-based inversion system present in this study could be a crucial part of such a system, enabling tracking air pollutant emissions by sector with low latency.

## CRediT authorship contribution statement

**Hui Li:** Formal Analysis, Investigation, Methodology, Software, Visualization, Writing - Original Draft, Writing - Review & Editing. **Bo Zheng:** Conceptualization, Funding Acquisition, Investigation, Methodology, Project Administration, Supervision, Writing - Review & Editing. **Yu Lei:** Writing - Review & Editing. **Didier Hauglustaine:** Software, Writing - Review & Editing. **Cuihong Chen:** Investigation. **Xin Lin:** Software, Writing - Review & Editing. **Yi Zhang:** Writing - Review & Editing. **Qiang Zhang:** Supervision, Writing - Review & Editing. **Kebin He:** Supervision, Writing - Review & Editing.

## Declaration of competing interest

The authors declare that they have no known competing financial interests or personal relationships that could have appeared to influence the work reported in this paper.

## Appendix A Supplementary data

Supporting Information.docx Supporting Information.docx
