# Supplementary material for: Trends and drivers of anthropogenic NOx emissions in China since 2020
Source: Environ Sci Ecotechnol. 2024 Apr 27;21:100425. doi: 10.1016/j.ese.2024.100425 (PMC11099326; doi:10.1016/j.ese.2024.100425)
Supplement: Supporting Information.docx [file mmc1.docx]

Supplementary Materials for

**Trends and drivers of anthropogenic NO*_x_* emissions in China since 2020**

Hui Li^1,2^, Bo Zheng^1,2*^ , Yu Lei^3^, Didier Hauglustaine^4^, Cuihong Chen^5^, Xin Lin^4^, Yi Zhang^6^, Qiang Zhang^7^, Kebin He^2,8^

^1^ Shenzhen Key Laboratory of Ecological Remediation and Carbon Sequestration, Institute of Environment and Ecology, Tsinghua Shenzhen International Graduate School, Tsinghua University, Shenzhen, 518055, China.

^2^ State Environmental Protection Key Laboratory of Sources and Control of Air Pollution Complex, Beijing, 100084, China.

^3^ Chinese Academy for Environmental Planning, Beijing, 100012, People’s Republic of China

^4^ Laboratoire des Sciences du Climat et de l’Environnement, LSCE/IPSL, CEA-CNRS-UVSQ, Université Paris-Saclay, Gif-sur-Yvette, France

^5^ Satellite Application Center for Ecology and Environment, MEE, Beijing, 100094, China.

^6^ Institute of Future Human Habitats, Tsinghua Shenzhen International Graduate School, Tsinghua University, Shenzhen, 518055, China.

^7^ Ministry of Education Key Laboratory for Earth System Modeling, Department of Earth System Science, Tsinghua University, Beijing, 100084, China.

^8^ State Key Joint Laboratory of Environment Simulation and Pollution Control, School of Environment, Tsinghua University, Beijing, 100084, China.

Corresponding author: Bo Zheng (bozheng@sz.tsinghua.edu.cn)

**Contents of this file**

- Materials and Methods
- Figures S1 to S10
- Tables S1 to S3

**Materials and Methods**

**1.1 The calculation of *β***

*β* is a unitless factor linking the changes in NO_2_ TVCDs to the changes in anthropogenic NO*_x_* emissions. To acquire this parameter, we conduct a perturbation simulation reducing 40% of China’s anthropogenic NO*_x_* emissions in 2019, and then calculate *β* as follows:

$$\beta_{t,i}=\frac{\Delta E_{t,i,\text{bottom-up},2019}}{E_{t,i,\text{bottom-up},2019}}\div\frac{\Omega_{t,i,\text{perturbed},2019}-\Omega_{t,i,\text{base},2019}}{\Omega_{t,i,\text{base},2019}}$$

Where *t* and *i* represent the month and grid cell (i.e., 0.5°×0.625°). Ω*_t,i_*_,perturbed,2019_ and Ω*_t,i_*_,base,2019_ are GEOS-Chem simulated NO_2_ TVCDs at the satellite overpass time under the perturbation and baseline simulation, respectively. ∆*E_t,i,_*_bottom-up,2019_/ *E_t,i,_*_bottom-up,2019_ is the 40% reduction in anthropogenic NO*_x_* emissions over China.

We have conducted a sensitivity test on the *β* value by introducing different perturbation intensities between 30-50%, which shows that the *β* value is not sensitive to emission perturbation with changes no more than 1.0% [1].

**1.2 The GEOS-Chem model simulation**

The atmospheric transport model we adopted is the GEOS-Chem 12.3.0 (<https://geoschem.github.io/>) driven by the meteorological fields from the MERRA-2 Reanalysis of the NASA Global Modeling and Assimilation Office [2]. To conduct the simulation over China, we set up the nested-grid configuration over Southeast Asia (0.5° × 0.625°) with boundary conditions acquired from a global simulation (2° × 2.5°). The chemical mechanism adopted is the "tropchem," which includes the full chemistry in the troposphere. The anthropogenic emissions over Southeast Asia are derived from the MIX inventory [3], in which the emissions over mainland China are substituted by the MEIC inventory [4, 5]. Natural emissions like lightning [6] and soil [7] are also included. Vertical mixing in the planetary boundary layer is simulated using a nonlocal mixing scheme [8].

The GEOS-Chem is mainly used to establish the relation between the changes in NO_2_ TVCDs and anthropogenic NO*_x_* emissions (*β* calculation) and exclude the meteorological annual variation-induced changes of NO_2_ TVCDs in this research. On the one hand, we compute *β* by introducing emission perturbations in the GEOS-Chem simulation on baseline year (i.e., 2019) as elaborated in Section 1.1. On the other hand, we quantify and remove the changes in NO_2_ TVCDs caused by the meteorological annual variation by conducting GEOS-Chem simulations with fixed emission input (2019 MEIC inventory) and meteorological data for the respective year (i.e., 2020, 2021, and 2022). Details are seen in Section 1.4.

**1.3 The sectoral attribution of satellite-constrained total NO*_x_* emissions**

We integrate top-down total NO*_x_* emissions and bottom-up sectoral profiles to infer the satellite-constrained sectoral NO*_x_* emissions. Initially, we calculate the difference between the bottom-up and top-down estimates in grid cells where a specific source sector dominates (contributing over 50% of emissions within a grid). These discrepancies serve to infer scaling factors for each sector, which are then applied to correct the bottom-up sectoral NO*_x_* emissions accordingly. Following this, we rescale the corrected bottom-up emissions to align with the satellite-constrained NO*_x_* emissions in terms of total emissions to derive the satellite-constrained sectoral NO*_x_* emissions (i.e., power, industry, transport, and residential sectors).

Where *t*, *s*, *i*, and *y* represent the month, sector, grid cell (i.e., 0.5°×0.625°), and year (i.e., 2020, 2021, and 2022), respectively. *E_t,i,_*_sate_*_,y_* is the anthropogenic NO*_x_* emissions constrained by satellite NO_2_ TVCDs (i.e., TROPOMI or OMI). *E_t,i,_*_bottom-up,2019_ is the updated NO*_x_* emissions in year *y* using the bottom-up method.

**1.4 The exclusion of** **meteorological variation-induced annual changes of NO_2_ TVCDs**

We first perform a baseline simulation for 2019 and then fix anthropogenic NO*_x_* emissions in 2019 and change the meteorological fields to 2020, 2021, and 2022, respectively. Then, the meteorological annual variation-induced changes of satellite NO_2_ are excluded as follows.

$$(\frac{\Delta\Omega}{\Omega})_{t,i,\text{anth},y}=\frac{\Omega_{t,i,\text{sate},y}-\Omega_{t,i,\text{sate},2019}}{\Omega_{t,i,\text{sate},2019}}-\frac{\Omega_{t,i,\text{simu\_fixemis},y}-\Omega_{t,i,\text{simu},2019}}{\Omega_{t,i,\text{simu},2019}}$$

Where *t*, *i*, and *y* represent the month, grid cell (i.e., 0.5°×0.625°), and year (i.e., 2020, 2021, and 2022), respectively. Ω*_t,i,_*_simu_fixemis_*_,y_* refers to the simulated NO_2_ TVCDs based on the fixed anthropogenic emissions in 2019. (Ω*_t,i,_*_simu_fixemis_*_,y_* - Ω*_t,i,_*_simu_*_,2019_*)/Ω*_t,i,_*_simu_*_,2019_* reflects the relative changes in NO_2_ TVCDs induced by the meteorological variations, and (Ω*_t,i,_*_sate_*_,y_* - Ω*_t,i,_*_sate_*_,2019_*)/Ω*_t,i,_*_sate_*_,2019_* represents the relative changes in satellite NO_2_ TVCDs from 2019 to year *y*. (ΔΩ/Ω)*_t,i,_*_anth_*_,y_* represents the relative changes in NO_2_ TVCDs due to anthropogenic emissions.

**1.5 The comparison between GEOS-Chem simulated and ground observational NO_2_ concentration**

Apart from comparing GEOC-Chem simulated NO_2_ TVCDs with satellite observational data, we extend the analysis to include a comparison with ground-based NO_2_ concentrations measured by surface stations. These ground-level measurements are obtained from the Chinese National Environmental Monitoring Center (CNEMC, http://www.cnemc.cn/). Recognizing a positive bias in surface NO_2_ measurements obtained via chemiluminescence analyzers, we have applied corrections to the GEOS-Chem simulated NO_2_ concentration based on the method from previous literature [9, 10]. The results, as depicted in Fig. S2, reveal a strong correlation between simulated NO_2_ and surface NO_2_ concentrations, providing additional evidence for the reliability and robustness of our findings. The low bias in GEOS-Chem simulated surface NO_2_ concentrations (with a slope in the range of 0.7-0.8 and an NMB (Normalized Mean Bias) around -40%) represents a common phenomenon due to the coarse spatial resolution of the GEOS-Chem model, which has been consistently reported by previous research [9, 11, 12].

**1.6 LMDz-INCA NO_2_ simulation with Carbon Monitor based NO*_x_* emissions**

(1) Carbon Monitor based NO*_x_* emission estimates

Given the constant CO_2_ emission factors, the annual fluctuations in sector-specific CO_2_ emissions from Carbon Monitor [13] (https://cn.carbonmonitor.org/) offer an alternative metric for gauging changes in fossil fuel consumption. Consequently, we employ these year-over-year changes in sector-specific CO_2_ emissions to update monthly NO*_x_* emissions in 2020 and 2021, starting from the baseline of 2019 MEIC [4]. This approach enables us to derive NO*_x_* emissions by solely accounting for variations in activity levels while keeping NO*_x_* emission factors unchanged. Therefore, its LMDz-INCA simulation facilitates an exploration of the trend in NO_2_ concentrations under the assumption of unchanged NO*_x_* emission factors over the years.

Where *t*, *s*, and *y* represent the month, sector, and year (i.e., 2020 and 2021), respectively. *E*_CO2_*_,t,s,_*_carbon_monitor_*_,y_* refers to the CO_2_ emissions from sector *s* in month *t* of year *y* derived from Carbon Monitor. *E*_CO2_*_,t,s,_*_carbon_monitor_*_,_*_2019_ refers to the CO_2_ emissions from sector *s* in month *t* of 2019 derived from Carbon Monitor. *E*_NOx_*_,t,s,_*_MEIC_*_,_*_2019_ is the NO*_x_* emissions from sector *s* in month *t* of 2019 from the MEIC emission inventory. *E*_NOx_*_,t,s,_*_anth_*_,y_* is the Carbon Monitor based NO*_x_* emissions of sector *s* in month *t* of target year *y*.

(2) LMDz-INCA NO_2_ simulation

We drive the global chemical transport model (LMDZ-INCA) [14] with Carbon Monitor based NO*_x_* emissions to simulate the corresponding NO_2_ TVCDs. The details of the model setting could be found in Peng, et al. [14]. The global anthropogenic emissions are acquired from the Community Emissions Data System (CEDS) emission inventory [15], while the NO*_x_* emissions over China are substituted by the Carbon Monitor-based NO*_x_* emissions from this study. The natural emissions, such as lightning NO*_x_* emissions, are also included. The meteorological fields used to drive the LMDZ-INCA model simulations are derived from the ECMWF ERA5 reanalysis dataset [16].

In addition to the Carbon Monitor based NO*_x_* emissions, we also utilize our estimated TROPOMI-constrained NO*_x_* emissions (from 2020 to 2022) to drive the LMDZ-INCA model. This is done to assess whether simulated NO_2_ TVCDs with our estimated emissions replicate the observed trend in NO_2_ concentrations.

The LMDz-INCA simulation results are depicted in Fig. S3b, where the light orange line represents the year-on-year changes in NO_2_ TVCDs simulated using Carbon Monitor-based NO*_x_* emissions, and the deep orange line signifies the year-on-year changes in NO_2_ TVCDs simulated with TROPOMI-constrained NO*_x_* emissions. It is evident that the latter successfully reproduces the annual trend observed in TROPOMI/OMI NO_2_ TVCDs, while the former, accounting only for changes in anthropogenic activity levels, fails to capture the decline in NO_2_ TVCDs in 2021.

**1.7 Annual NO_2_ concentration changes measured by ground stations in China**

The Ministry of Ecology and Environment of China releases an annual report at the beginning of each year, summarizing the ambient air quality nationwide in the previous year. This report includes the annual average concentration of NO_2_ measured by about 2000 ground stations across the country. Based on these releases, China’s surface NO_2_ concentration are 24 μg m^-3^ and 21 μg m^-3^ in 2020 (<https://www.mee.gov.cn/xxgk2018/xxgk/xxgk15/202101/t20210115_817499.html>) and 2022 (https://www.mee.gov.cn/ywdt/xwfb/202301/t20230128_1014006.shtml), respectively. Based on these data, we calculate a 12.5% decrease in surface NO_2_ concentrations in China from 2020 to 2022.


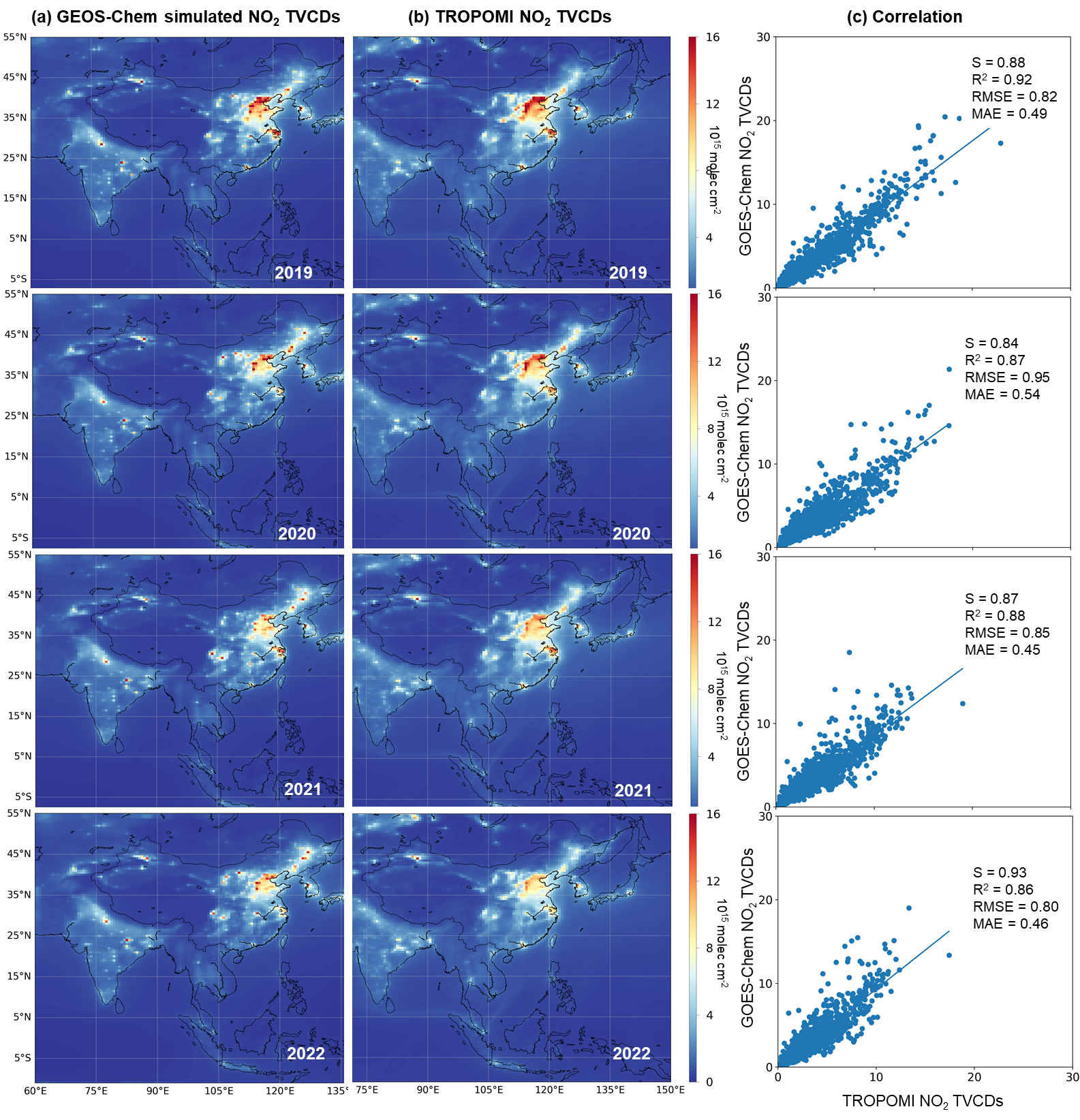


**Figure S1. Comparison of the TROPOMI observational and simulated NO_2_ TVCDs in 2019, 2020, 2021, and 2022.** Column (a) and (b) present the spatial distribution of yearly averaged NO_2_ TVCDs (10^15^ molec cm^-2^) from GEOS-Chem simulation and TROPOMI in 2019, 2020, 2021, and 2022, respectively (from top to bottom). Column (c) shows the scatter plots between model simulations and TROPOMI retrievals of yearly national NO_2_ TVCDs in 2019, 2020, 2021, and 2022, respectively (from top to bottom) with regression slope (S), correlation coefficient (R^2^), RMSE (root mean square error) (10^15^ molec cm^-2^), and MAE (mean absolute error) (10^15^ molec cm^-2^) displaced in the corresponding panel.


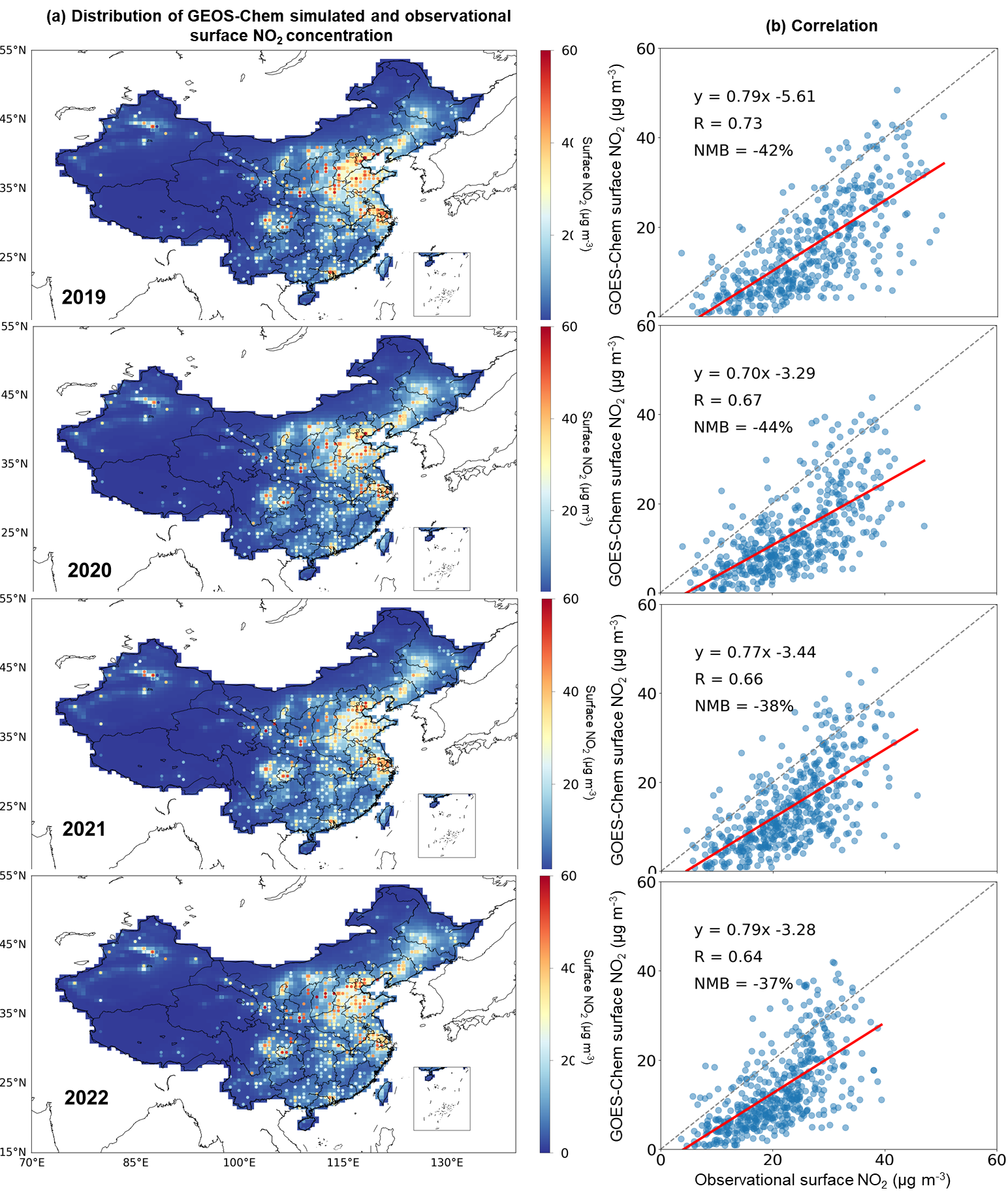


**Figure S2. Comparison of the surface observational and simulated NO_2_ concentration in 2019, 2020, 2021, and 2022 (from top to bottom).** Column (a) shows the spatial distribution of GEOS-Chem simulated and surface-monitored NO_2_ concentration (μg m^-3^). Column (b) presents the correlation between GEOS-Chem simulated and surface-monitored NO_2_ concentration (μg m^-3^). The points in the map in Column (a) refer to the average surface NO_2_ concentration measured by the ground station within each grid, and the points in the Column (b) represent the averaged surface NO_2_ concentration within each grid that is simultaneously measured by the surface station and simulated by GEOS-Chem.


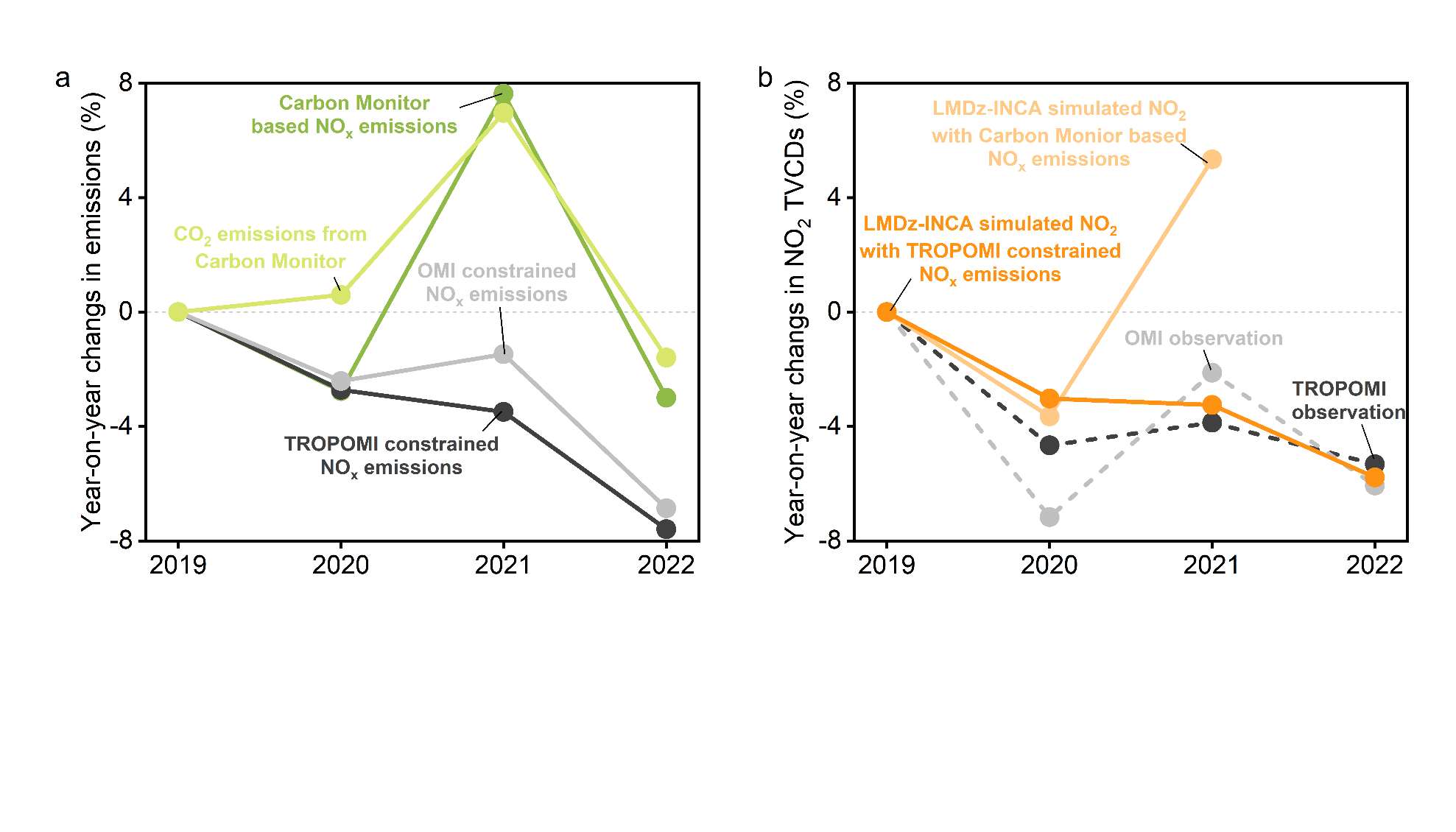


**Figure S3. Comparison of year-on-year changes in (a) NO*_x_* emissions and (b) NO_2_ columns between 2020 and 2022.** The light green line in (a) refers to the CO_2_ emissions from Carbon Monitor (https://cn.carbonmonitor.org/). The deep green line in (a) refers to the changes in NO*_x_* emissions estimated based on the changes in sectoral CO_2_ emissions from Carbon Monitor (correspond to the Carbon Monitor-based NO*_x_* emissions in the manuscript). The LMDz-INCA simulated NO_2_ with TROPOMI constrained NO*_x_* emissions (solid deep orange line in (b)) refer to the simulated NO_2_ TVCDs using our inversion-based NO*_x_* emissions with TROPOMI. The LMDz-INCA simulated NO_2_ with Carbon Monitor based NO*_x_* emissions (solid light orange line in (b)) refer to the simulated NO_2_ TVCDs using Carbon Monitor-based NO*_x_* emissions.


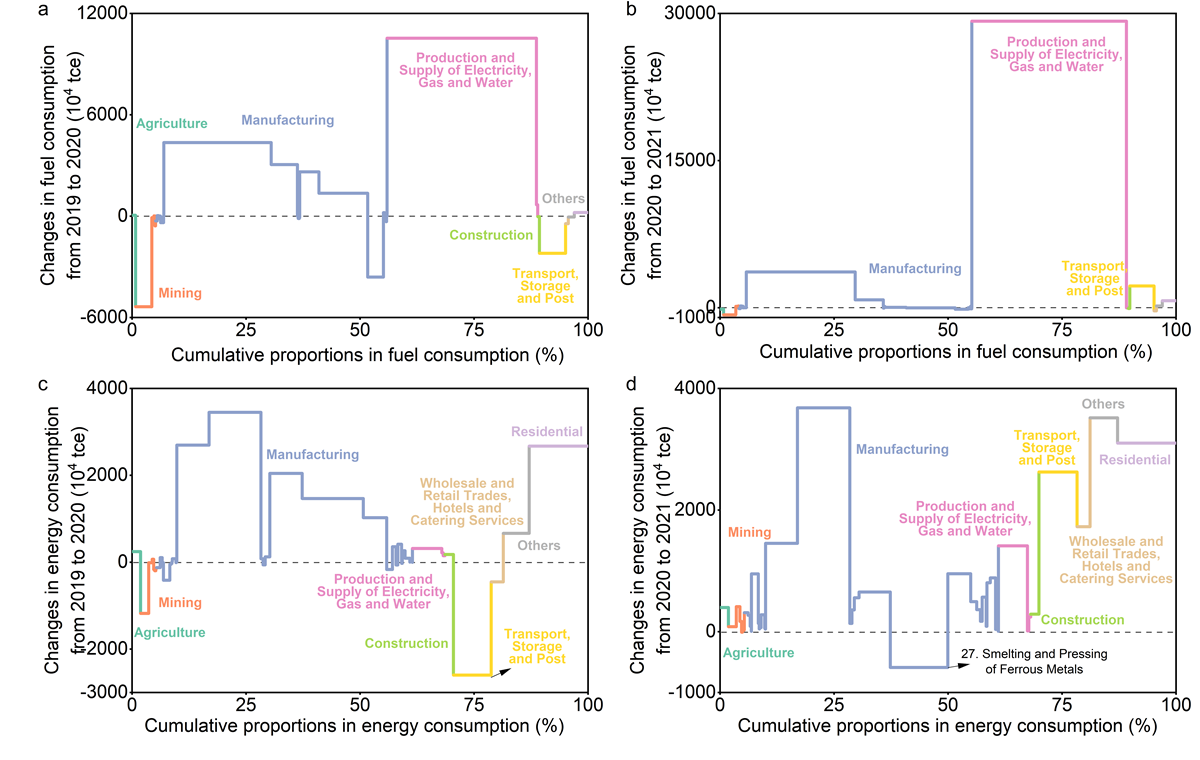


**Figure S4. Annual changes in sectoral fossil fuel consumption and total energy consumption between 2020 and 2021**. (a) and (b) show the changes in sectoral fossil fuel consumption from 2019 to 2020 and from 2020 to 2021, respectively. (c) and (d) show the changes in sectoral total energy consumption from 2019 to 2020 and from 2020 to 2021, respectively. Color corresponds to the industrial category (detailed information on industrial categories is seen in Table S1). The sectoral fossil fuel consumption and total energy consumption in 2022 is currently unavailable, thus it is not presented here. The x-axis in (a) and (b) correspond to the third and fourth columns in Table S1, and the x-axis in (c) and (d) correspond to the third and fourth columns in Table S2. The numbers within sectors correspond to the number of categories in Table S1.


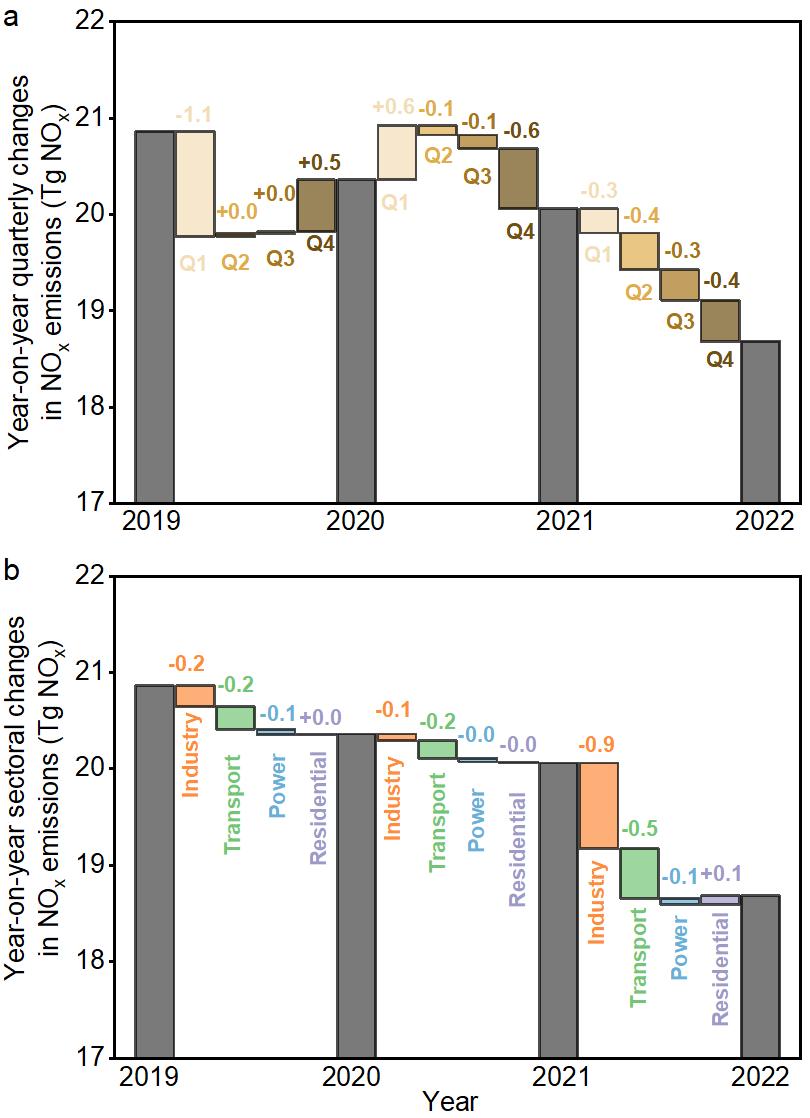


**Figure S5.** **Year-on-year NO*_x_* emission variations by quarter and sector between 2020 and 2022 constrained by OMI**. (a) shows the year-on-year quarterly NO*_x_* emission variations between 2020 and 2022. (b) shows the year-on-year sectoral NO*_x_* emission variations between 2020 and 2022.


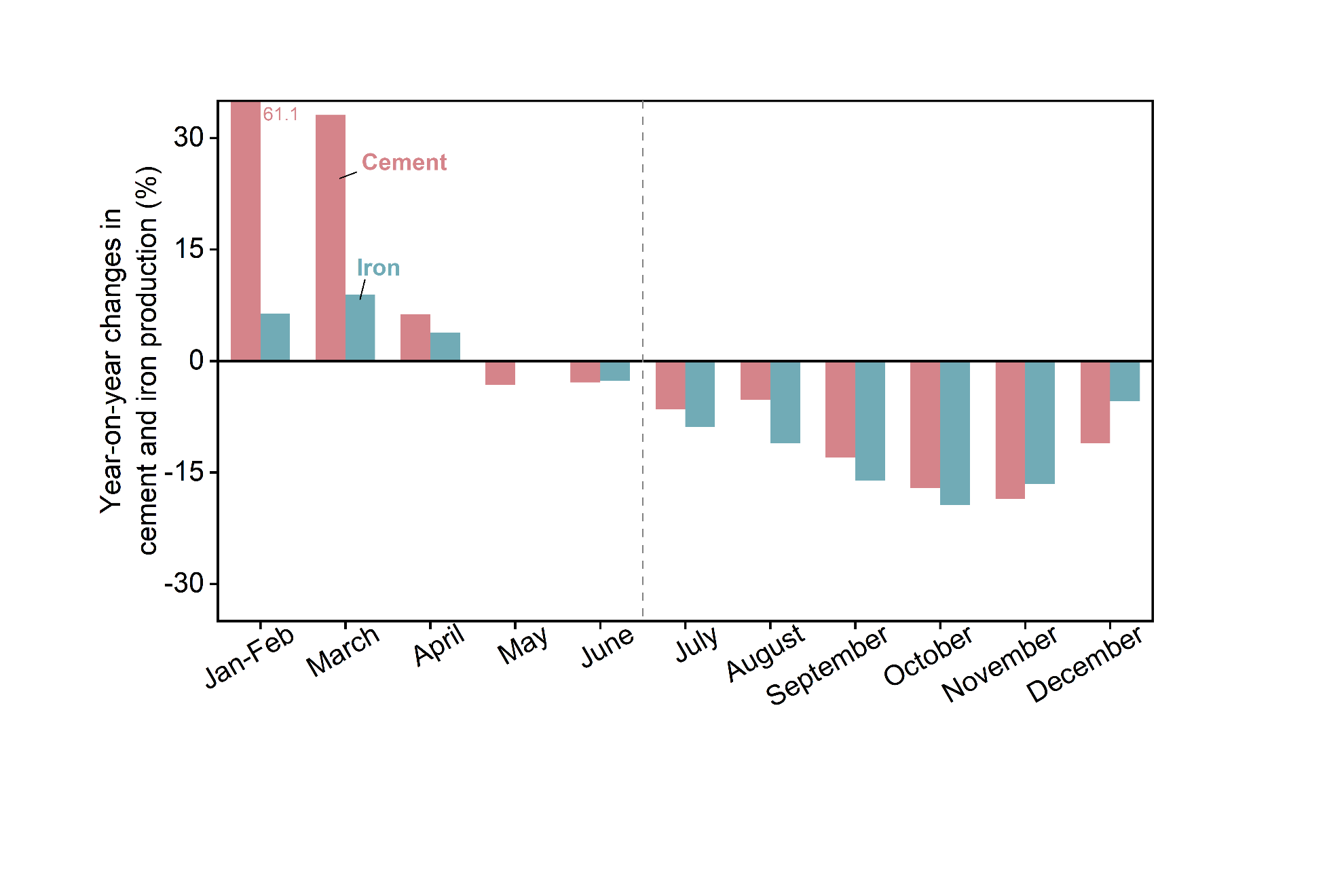


**Figure S6. Year-on-year changes in monthly cement and iron production in 2021**. Red and blue columns refer to the cement and iron production, respectively.


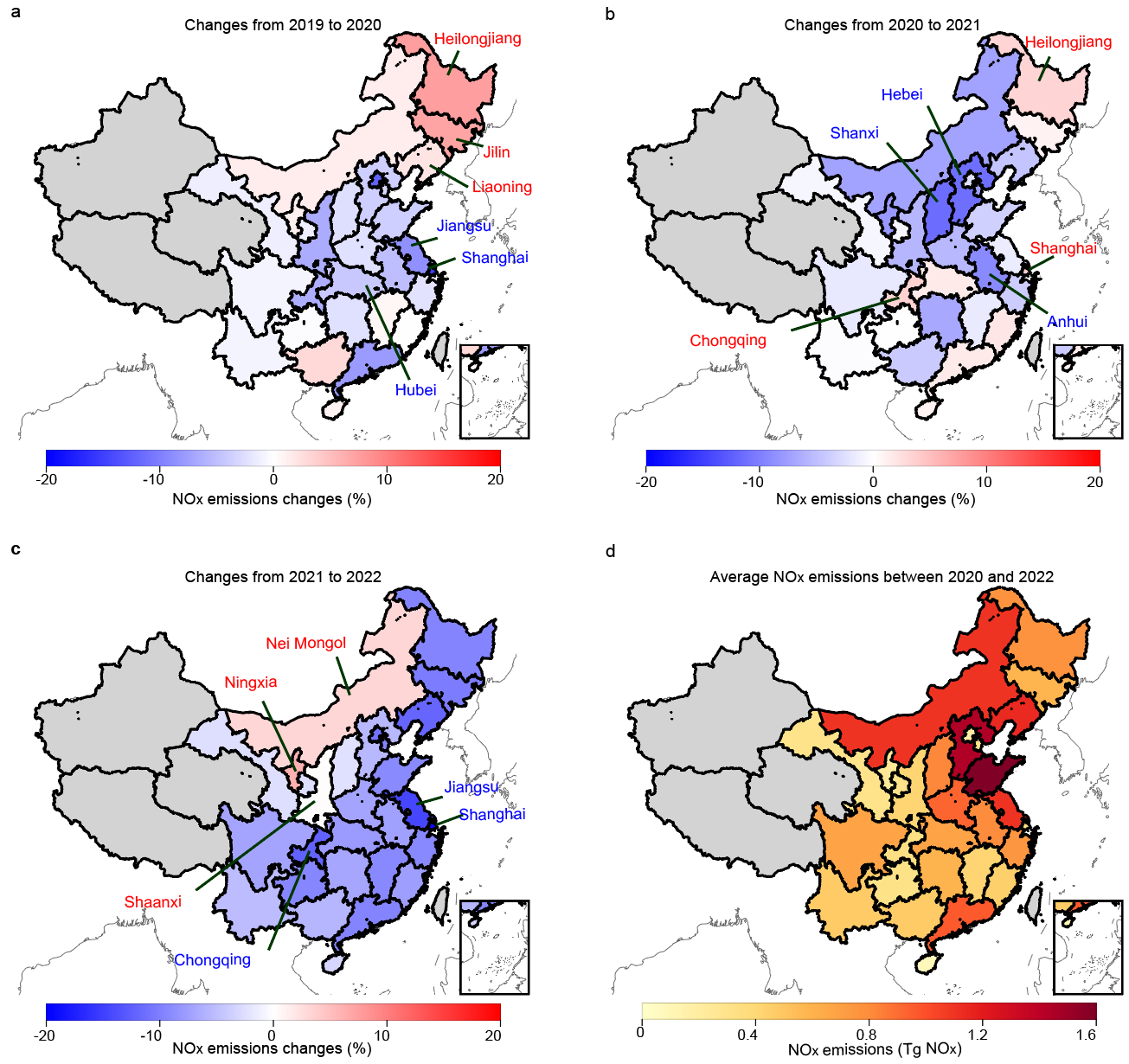


**Figure S7.** **Year-on-year changes in provincial NO*_x_* emissions between 2020 and 2022**. (a), (b), and (c) present annual changes in provincial NO*_x_* emissions from 2019 to 2022. (d) shows annual average provincial NO*_x_* emissions between 2020 and 2022. Only provinces with enough TROPOMI observations that covered more than 90% of anthropogenic NO*_x_* emissions are shown here; otherwise, they are gray-shaded.


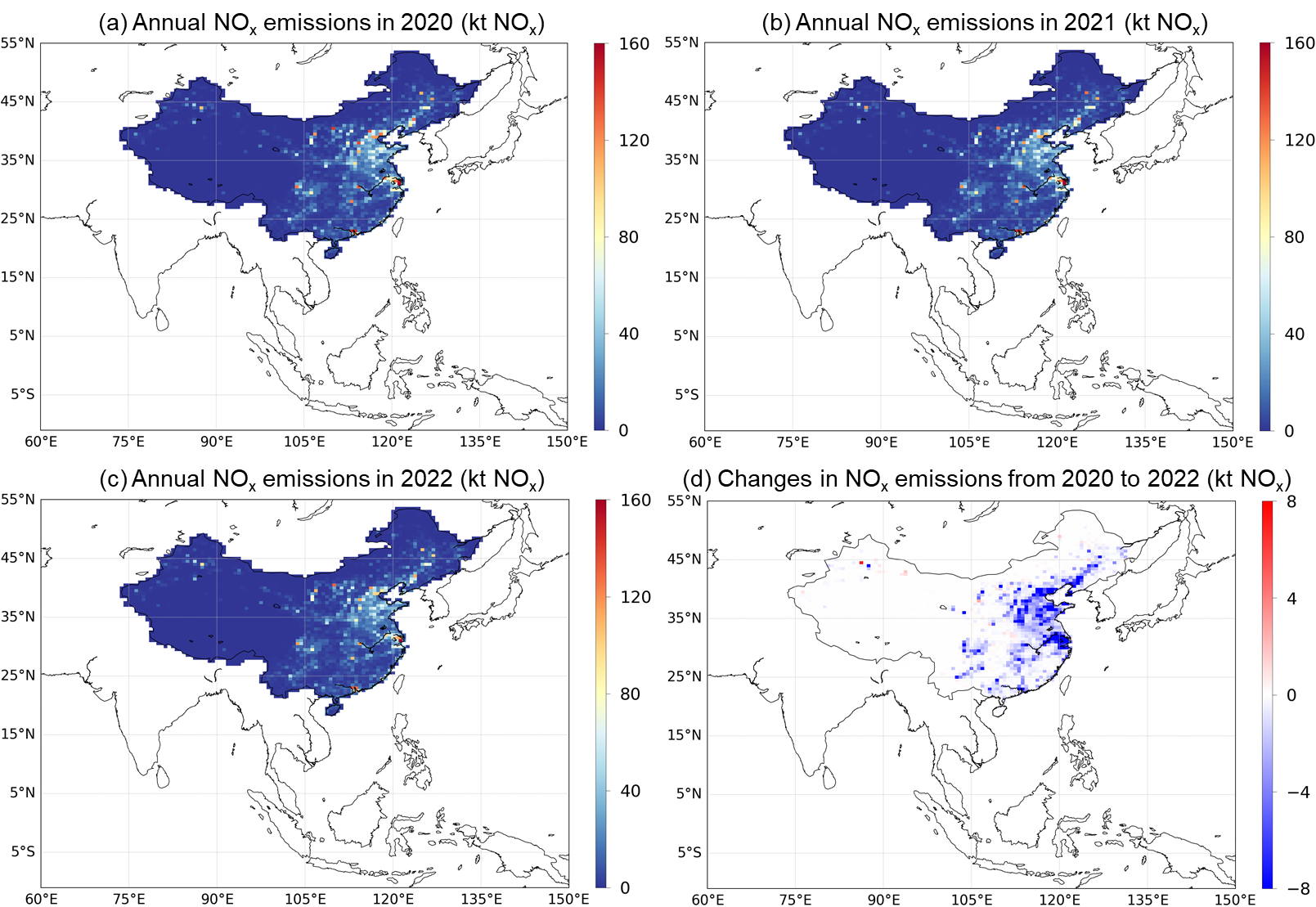


**Figure S8.** **Comparison of the spatial distribution of annual NO*_x_* emissions (0.5°×0.625°) from 2020 to 2022**. (a), (b), and (c) present annual NO*_x_* emissions in 2020, 2021, and 2022. (d) shows the changes in NO*_x_* emissions from 2020 to 2022.


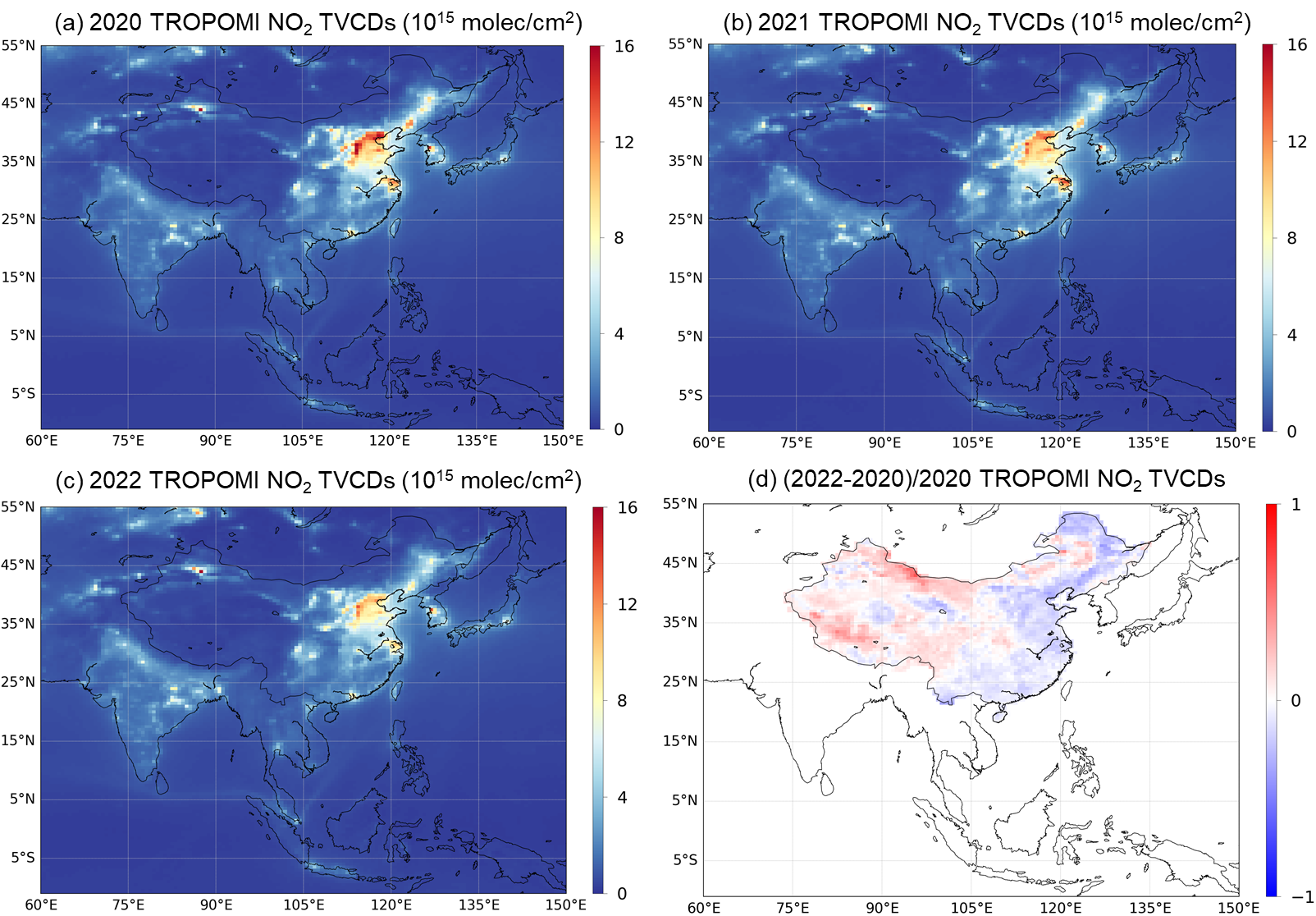


**Figure S9.** **Comparison of the spatial distribution of annual average NO_2_ TVCDs (0.5°×0.625°) from 2020 to 2022**. (a), (b), and (c) present annual average NO_2_ TVCDs in 2020, 2021, and 2022. (d) shows the changes in NO_2_ TVCDs from 2020 to 2022.


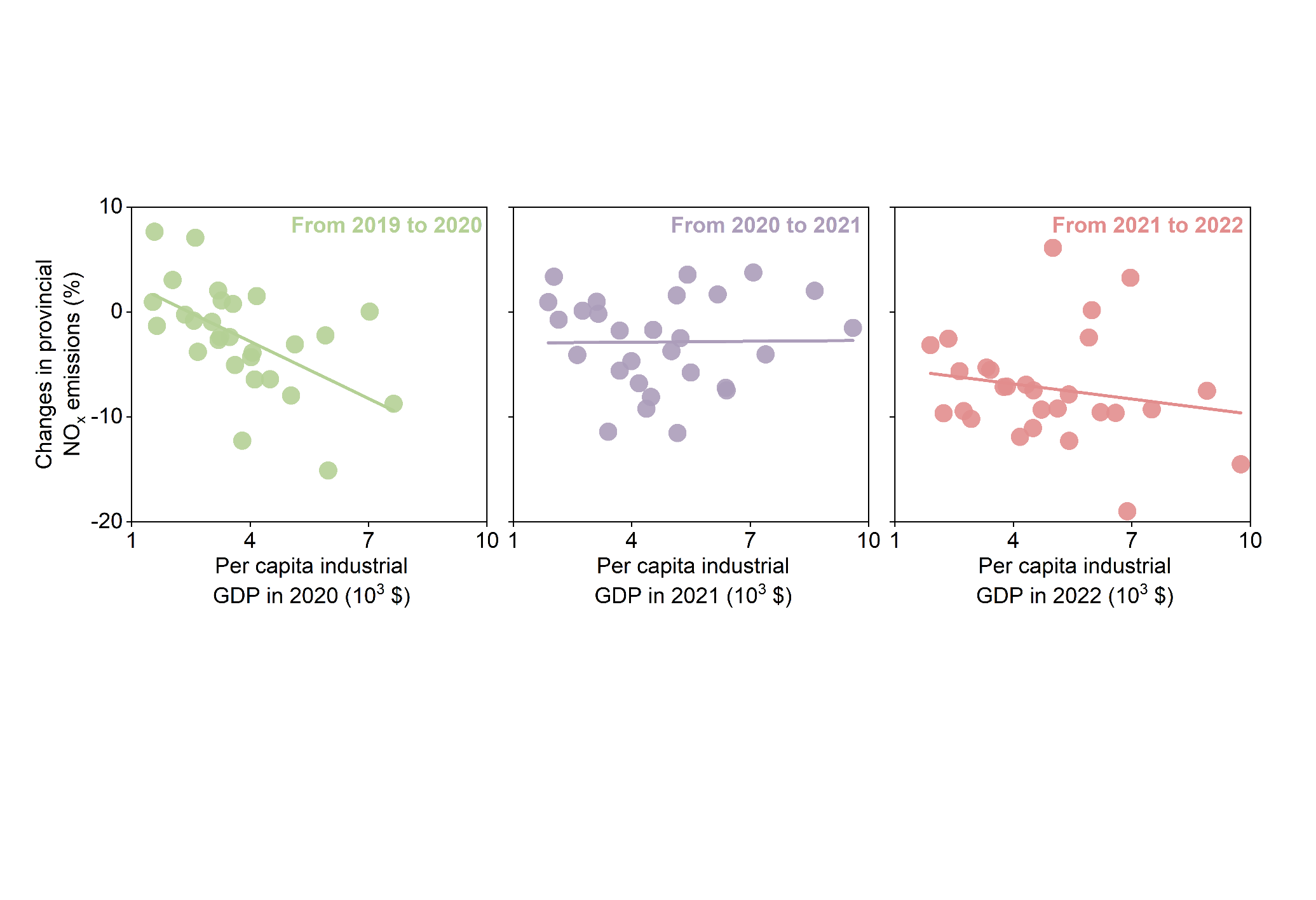


**Figure S10. Correlation between the annual changes in provincial NO*_x_* emissions and provincial per capita industrial gross domestic product (GDP) in 2020, 2021, and 2022 (from left to right).**

**Table S1**. Categories of industry sectors and production sectors in this study and the sectoral proportion of fossil fuel consumption in 2019, 2020, and 2021.

| **Industry sector** | **Production sector number** | **Proportion in 2019 (%)** | **Proportion in 2020 (%)** | **Proportion in 2021 (%)** |
| --- | --- | --- | --- | --- |
| A. Agriculture | 1. Agriculture, Forestry, Animal Husbandry and Fishery | 0.76 | 0.76 | 0.69 |
| B. Mining | 2. Mining and Washing of Coal | 3.58 | 2.70 | 2.44 |
|  | 3. Extraction of Petroleum and Natural Gas | 0.47 | 0.45 | 0.45 |
|  | 4. Mining and Processing of Ferrous Metal Ores | 0.06 | 0.07 | 0.09 |
|  | 5. Mining and Processing of Non-Ferrous Metal Ores | 0.02 | 0.02 | 0.02 |
|  | 6. Mining and Processing of Nonmetal Ores | 0.13 | 0.10 | 0.12 |
|  | 7. Professional and Support Activities for Mining | 0.13 | 0.04 | 0.04 |
|  | 8. Mining of Other Ores | 0.00 | 0.00 | 0.00 |
| C. Manufacturing | 9. Processing of Food from Agricultural Products | 0.36 | 0.31 | 0.28 |
|  | 10. Manufacture of Foods | 0.31 | 0.31 | 0.32 |
|  | 11. Manufacture of Liquor, Beverages and Refined Tea | 0.13 | 0.11 | 0.11 |
|  | 12. Manufacture of Tobacco | 0.00 | 0.00 | 0.00 |
|  | 13. Manufacture of Textile | 0.21 | 0.19 | 0.20 |
|  | 14. Manufacture of Textile, Wearing Apparel and Accessories | 0.03 | 0.03 | 0.03 |
|  | 15. Manufacture of Leather, Fur, Feather and Related Products and Footwear | 0.01 | 0.01 | 0.01 |
|  | 16. Processing of Timber, Manufacture of Wood, Bamboo, Rattan, Palm, and Straw Products | 0.02 | 0.01 | 0.01 |
|  | 17. Manufacture of Furniture | 0.00 | 0.00 | 0.00 |
|  | 18. Manufacture of Paper and Paper Products | 0.67 | 0.60 | 0.57 |
|  | 19. Printing and Reproduction of Recording Media | 0.02 | 0.02 | 0.02 |
|  | 20. Manufacture of Articles for Culture, Education, Arts and Crafts, Sport and Entertainment Activities | 0.03 | 0.03 | 0.03 |
|  | 21. Processing of Petroleum, Coal and Other Fuels | 23.54 | 23.88 | 23.14 |
|  | 22. Manufacture of Raw Chemical Materials and Chemical Products | 5.77 | 6.16 | 5.95 |
|  | 23. Manufacture of Medicines | 0.16 | 0.15 | 0.14 |
|  | 24. Manufacture of Chemical Fibers | 0.24 | 0.21 | 0.22 |
|  | 25. Manufacture of Rubber and Plastics Products | 0.10 | 0.09 | 0.09 |
|  | 26. Manufacture of Non-metallic Mineral Products | 4.21 | 4.55 | 4.32 |
|  | 27. Smelting and Pressing of Ferrous Metals | 10.69 | 10.75 | 10.18 |
|  | 28. Smelting and Pressing of Non-ferrous Metals | 3.45 | 2.84 | 2.67 |
|  | 29. Manufacture of Metal Products | 0.25 | 0.28 | 0.27 |
|  | 30. Manufacture of General Purpose Machinery | 0.09 | 0.11 | 0.09 |
|  | 31. Manufacture of Special Purpose Machinery | 0.05 | 0.04 | 0.04 |
|  | 32. Manufacture of Automobiles | 0.08 | 0.08 | 0.08 |
|  | 33. Manufacture of Railway, Ship, Aerospace and Other Transport Equipment | 0.05 | 0.05 | 0.06 |
|  | 34. Manufacture of Electrical Machinery and Apparatus | 0.03 | 0.04 | 0.06 |
|  | 35. Manufacture of Computers, Communication and Other Electronic Equipment | 0.13 | 0.08 | 0.07 |
|  | 36. Manufacture of Measuring Instruments and Machinery | 0.00 | 0.00 | 0.00 |
|  | 37. Other Manufacture | 0.01 | 0.01 | 0.01 |
|  | 38. Utilization of Waste Resources | 0.07 | 0.09 | 0.07 |
|  | 39. Repair Service of Metal Products, Machinery and Equipment | 0.00 | 0.00 | 0.00 |
| D. Production and Supply of Electricity, Gas and Water | 40. Production and Supply of Electric Power and Heat Power | 32.77 | 33.93 | 36.42 |
|  | 41. Production and Supply of Gas | 0.28 | 0.38 | 0.36 |
|  | 42. Production and Supply of Water | 0.01 | 0.01 | 0.01 |
| E. Construction | 43. Construction | 0.36 | 0.35 | 0.32 |
| F. Transport, Storage and Post | 44. Transport, Storage and Post | 5.73 | 5.30 | 5.34 |
| G. Wholesale and Retail Trades, Hotels and Catering Services | 45. Wholesale and Retail Trades, Hotels and Catering Services | 0.62 | 0.54 | 0.46 |
| H. Others | 46. Others | 1.30 | 1.28 | 1.23 |
| I. Residential | 47. Residential | 3.03 | 3.02 | 2.96 |

*The third and fourth columns correspond to the x-axis in Fig. S4(a) and Fig. S4(b), respectively, illustrating the sectoral proportion of fossil fuel consumption in 2019 and 2020.

**Table S2**. Categories of industry sectors and production sectors in this study and the sectoral proportion of total energy consumption in 2019, 2020, and 2021.

| **Industry sector** | **Production sector number** | **Proportion in 2019 (%)** | **Proportion in 2020 (%)** | **Proportion in 2021 (%)** |
| --- | --- | --- | --- | --- |
| A. Agriculture | 1. Agriculture, Forestry, Animal Husbandry and Fishery | 1.85 | 1.86 | 1.84 |
| B. Mining | 2. Mining and Washing of Coal | 2.08 | 1.80 | 1.72 |
|  | 3. Extraction of Petroleum and Natural Gas | 0.77 | 0.75 | 0.79 |
|  | 4. Mining and Processing of Ferrous Metal Ores | 0.34 | 0.35 | 0.36 |
|  | 5. Mining and Processing of Non-Ferrous Metal Ores | 0.26 | 0.22 | 0.21 |
|  | 6. Mining and Processing of Nonmetal Ores | 0.27 | 0.23 | 0.24 |
|  | 7. Professional and Support Activities for Mining | 0.10 | 0.06 | 0.06 |
|  | 8. Mining of Other Ores | 0.13 | 0.10 | 0.10 |
| C. Manufacturing | 9. Processing of Food from Agricultural Products | 0.85 | 0.80 | 0.82 |
|  | 10. Manufacture of Foods | 0.42 | 0.43 | 0.46 |
|  | 11. Manufacture of Liquor, Beverages and Refined Tea | 0.26 | 0.24 | 0.24 |
|  | 12. Manufacture of Tobacco | 0.04 | 0.04 | 0.04 |
|  | 13. Manufacture of Textile | 1.52 | 1.40 | 1.51 |
|  | 14. Manufacture of Textile, Wearing Apparel and Accessories | 0.19 | 0.17 | 0.19 |
|  | 15. Manufacture of Leather, Fur, Feather and Related Products and Footwear | 0.11 | 0.10 | 0.10 |
|  | 16. Processing of Timber, Manufacture of Wood, Bamboo, Rattan, Palm, and Straw Products | 0.21 | 0.20 | 0.22 |
|  | 17. Manufacture of Furniture | 0.08 | 0.08 | 0.09 |
|  | 18. Manufacture of Paper and Paper Products | 0.79 | 0.79 | 0.80 |
|  | 19. Printing and Reproduction of Recording Media | 0.10 | 0.10 | 0.11 |
|  | 20. Manufacture of Articles for Culture, Education, Arts and Crafts, Sport and Entertainment Activities | 0.10 | 0.10 | 0.10 |
|  | 21. Processing of Petroleum, Coal and Other Fuels | 6.68 | 7.08 | 6.98 |
|  | 22. Manufacture of Raw Chemical Materials and Chemical Products | 10.93 | 11.38 | 11.49 |
|  | 23. Manufacture of Medicines | 0.45 | 0.45 | 0.46 |
|  | 24. Manufacture of Chemical Fibers | 0.50 | 0.47 | 0.52 |
|  | 25. Manufacture of Rubber and Plastics Products | 1.00 | 1.00 | 1.05 |
|  | 26. Manufacture of Non-metallic Mineral Products | 6.84 | 7.10 | 6.85 |
|  | 27. Smelting and Pressing of Ferrous Metals | 13.41 | 13.42 | 12.60 |
|  | 28. Smelting and Pressing of Non-ferrous Metals | 5.01 | 5.11 | 5.02 |
|  | 29. Manufacture of Metal Products | 1.34 | 1.28 | 1.31 |
|  | 30. Manufacture of General Purpose Machinery | 0.74 | 0.80 | 0.83 |
|  | 31. Manufacture of Special Purpose Machinery | 0.39 | 0.37 | 0.38 |
|  | 32. Manufacture of Automobiles | 0.75 | 0.82 | 0.88 |
|  | 33. Manufacture of Railway, Ship, Aerospace and Other Transport Equipment | 0.17 | 0.17 | 0.17 |
|  | 34. Manufacture of Electrical Machinery and Apparatus | 0.58 | 0.62 | 0.74 |
|  | 35. Manufacture of Computers, Communication and Other Electronic Equipment | 1.03 | 1.03 | 1.14 |
|  | 36. Manufacture of Measuring Instruments and Machinery | 0.05 | 0.05 | 0.06 |
|  | 37. Other Manufacture | 0.37 | 0.37 | 0.42 |
|  | 38. Utilization of Waste Resources | 0.13 | 0.14 | 0.14 |
|  | 39. Repair Service of Metal Products, Machinery and Equipment | 0.01 | 0.01 | 0.01 |
| D. Production and Supply of Electricity, Gas and Water | 40. Production and Supply of Electric Power and Heat Power | 6.51 | 6.44 | 6.37 |
|  | 41. Production and Supply of Gas | 0.27 | 0.31 | 0.29 |
|  | 42. Production and Supply of Water | 0.36 | 0.39 | 0.41 |
| E. Construction | 43. Construction | 1.88 | 1.87 | 1.83 |
| F. Transport, Storage and Post | 44. Transport, Storage and Post | 9.01 | 8.29 | 8.35 |
| G. Wholesale and Retail Trades, Hotels and Catering Services | 45. Wholesale and Retail Trades, Hotels and Catering Services | 2.79 | 2.64 | 2.83 |
| H. Others | 46. Others | 5.66 | 5.67 | 6.04 |
| I. Residential | 47. Residential | 12.66 | 12.92 | 12.83 |

*The third and fourth columns correspond to the x-axis in Fig. S4(c) and Fig. S4(d), respectively, illustrating the sectoral proportion of total energy consumption in 2019 and 2020.

**Table S3**. Provinces included in each region in Figure 4.

| **Region** | **Provinces** |
| --- | --- |
| North China | Beijing, Tianjin, Hebei, Shanxi, Nei Mongol |
| Northeast China | Heilongjiang, Jilin, Liaoning |
| East China | Shanghai, Jiangsu, Zhejiang, Anhui, Fujian, Jiangxi, Shandong |
| Central China | Henan, Hubei, Hunan |
| South China | Guangdong, Guangxi, Hainan |
| Southwest region | Chongqing, Sichuan, Guizhou, Yunnan |
| Northwest China | Shaanxi, Gansu, Ningxia |

**References**

[1] Zheng B, Geng G, Ciais P, et al. Satellite-based estimates of decline and rebound in China's CO_2_ emissions during COVID-19 pandemic. Science Advances, 2020, 6: eabd4998

[2] Rienecker MM, Suarez MJ, Gelaro R, et al. MERRA: NASA’s Modern-Era Retrospective Analysis for Research and Applications. Journal of Climate, 2011, 24: 3624-3648

[3] Li M, Zhang Q, Kurokawa JI, et al. MIX: a mosaic Asian anthropogenic emission inventory under the international collaboration framework of the MICS-Asia and HTAP. Atmos Chem Phys, 2017, 17: 935-963

[4] Zheng B, Zhang Q, Geng G, et al. Changes in China's anthropogenic emissions and air quality during the COVID-19 pandemic in 2020. Earth Syst Sci Data, 2021, 13: 2895-2907

[5] Zheng B, Tong D, Li M, et al. Trends in China's anthropogenic emissions since 2010 as the consequence of clean air actions. Atmos Chem Phys, 2018, 18: 14095-14111

[6] Murray LT, Jacob DJ, Logan JA, et al. Optimized regional and interannual variability of lightning in a global chemical transport model constrained by LIS/OTD satellite data. Journal of Geophysical Research Atmospheres, 2012, 117:

[7] Hudman RC, Moore NE, Mebust AK, et al. Steps towards a mechanistic model of global soil nitric oxide emissions: implementation and space based-constraints. Atmos Chem Phys, 2012, 12: 7779-7795

[8] Lin J-T, McElroy MB. Impacts of boundary layer mixing on pollutant vertical profiles in the lower troposphere: Implications to satellite remote sensing. Atmospheric Environment, 2010, 44: 1726-1739

[9] Qu Z, Henze DK, Cooper OR, et al. Impacts of global NO_x_ inversions on NO_2_ and ozone simulations. Atmos Chem Phys, 2020, 20: 13109-13130

[10] Lamsal LN, Martin RV, van Donkelaar A, et al. Ground-level nitrogen dioxide concentrations inferred from the satellite-borne Ozone Monitoring Instrument. Journal of Geophysical Research: Atmospheres, 2008, 113:

[11] Qu Z, Henze DK, Worden HM, et al. Sector-Based Top-Down Estimates of NO_x_, SO_2_, and CO Emissions in East Asia. Geophysical Research Letters, 2022, 49: e2021GL096009

[12] Laughner JL, Zare A, Cohen RC. Effects of daily meteorology on the interpretation of space-based remote sensing of NO_2_. Atmos Chem Phys, 2016, 16: 15247-15264

[13] Liu Z, Ciais P, Deng Z, et al. Carbon Monitor, a near-real-time daily dataset of global CO_2_ emission from fossil fuel and cement production. Scientific Data, 2020, 7: 392

[14] Peng S, Lin X, Thompson RL, et al. Wetland emission and atmospheric sink changes explain methane growth in 2020. Nature, 2022, 612: 477-482

[15] Hoesly RM, Smith SJ, Feng L, et al. Historical (1750–2014) anthropogenic emissions of reactive gases and aerosols from the Community Emissions Data System (CEDS). Geosci Model Dev, 2018, 11: 369-408

[16] Hersbach H, Bell B, Berrisford P, et al. The ERA5 global reanalysis. Quarterly Journal of the Royal Meteorological Society, 2020, 146: 1999-2049
